# The Binding Effect of Proteins on Medications and Its Impact on Electrochemical Sensing: Antipsychotic Clozapine as a Case Study

**DOI:** 10.3390/ph10030069

**Published:** 2017-08-01

**Authors:** George E. Banis, Thomas Winkler, Patricia Barton, Sheryl E. Chocron, Eunkyoung Kim, Deanna L. Kelly, Gregory F. Payne, Hadar Ben-Yoav, Reza Ghodssi

**Affiliations:** 1 Department of Bioengineering, University of Maryland, 2201 J.M. Patterson Hall, College Park, MD 20742, USA; gbanis@umd.edu (G.E.B.); winklert@kth.se (T.W.); pbarton@umd.edu (P.B.); sherylchb@gmail.com (S.E.C.); 2 Institute for Bioscience and Biotechnology Research, University of Maryland, Suite 5115 Plant Sciences Building, College Park, MD 20742, USA; kimk@ibbr.umd.edu (E.K.); gpayne@umd.edu (G.F.P.); 3 Maryland Psychiatric Research Center, University of Maryland School of Medicine, 655 W. Baltimore Street, Baltimore, MD 21201, USA; dkelly@mprc.umaryland.edu; 4 Department of Biomedical Engineering, Ben-Gurion University of the Negev, Beer Sheva 8410501, Israel; benyoav@bgu.ac.il; 5 Institute for Systems Research, University of Maryland, 2173 A.V. Williams Building, College Park, MD 20742, USA

**Keywords:** electrochemistry, clozapine, albumin, alpha-1 acid-glycoprotein, ultrafiltration

## Abstract

Clozapine (CLZ), a dibenzodiazepine, is demonstrated as the optimal antipsychotic for patients suffering from treatment-resistant schizophrenia. Like many other drugs, understanding the concentration of CLZ in a patient’s blood is critical for managing the patients’ symptoms, side effects, and overall treatment efficacy. To that end, various electrochemical techniques have been adapted due to their capabilities in concentration-dependent sensing. An open question associated with electrochemical CLZ monitoring is whether drug–protein complexes (i.e., CLZ bound to native blood proteins, such as serum albumin (SA) or alpha-1 acid-glycoprotein (AAG)) contribute to electrochemical redox signals. Here, we investigate CLZ-sensing performance using fundamental electrochemical methods with respect to the impact of protein binding. Specifically, we test the activity of bound and free fractions of a mixture of CLZ and either bovine SA or human AAG. Results suggest that bound complexes do not significantly contribute to the electrochemical signal for mixtures of CLZ with AAG or SA. Moreover, the fraction of CLZ bound to protein is relatively constant at 31% (AAG) and 73% (SA) in isolation with varying concentrations of CLZ. Thus, electrochemical sensing can enable direct monitoring of only the unbound CLZ, previously only accessible via equilibrium dialysis. The methods utilized in this work offer potential as a blueprint in developing electrochemical sensors for application to other redox-active medications with high protein binding more generally. This demonstrates that electrochemical sensing can be a new tool in accessing information not easily available previously, useful toward optimizing treatment regimens.

## 1. Introduction

Therapeutic drug monitoring (TDM) refers to measuring the presence of pharmaceutical substances, specifically those with a known therapeutic range, under physiological circulation [1]. TDM is necessary for effective treatment or management of a medical condition over time, and to at the same time minimize the adverse effects of the medication [2]. Effective TDM, however, is complicated by the presence of interfering species in serum. These species can include cells, proteins, amino acids, nucleotides, antioxidants, vitamins, etc. [3]. Proteins are of particular interest, since interactions with the medication can result in bound protein–drug complexes.

Traditional TDM employs liquid chromatography followed by mass spectrometry (LC/MS), which relies on physical separation of chemical species to obtain structural identities. The equipment required for MS is fairly expensive and only accessible to laboratories with the appropriate facilities. Typically, this technique is limited to assessing the total drug concentration, as the organic solvents employed denature proteins that may have bound to the drug. Assessing the individual (free and protein-bound) fractions requires additional sample pretreatment, e.g., in the form of equilibrium dialysis, and is rarely carried out. Recently, additional sensing methods have been under development that aim to provide the same information at only a fraction of the original requirements. In particular, electrochemical sensors have found widespread use in biomedical applications for concentration-dependent detection and quantification of analytes in the blood. 

Numerous proteins are present in concentrations greater than that of the target drug, yielding significant noise in measured electrochemical redox signals. Additionally, during electrochemical sensing, it is unclear as to whether or not the proteins react with the electrodes as well, a phenomena referred to as fouling [4]. This challenge is further compounded by the occurrence of cross-reactivity between these interferents and the drug, which may form byproducts that experience a shifted anodic potential relative to the reactants [5,6,7]. Several groups have reported the interfering impact of bovine serum albumin (BSA) on the detection of other electrochemically active species, such as porphyrin or thiamin [8,9]. Therefore, complex signal isolation, filtering and processing are useful in decreasing the background to obtain more information that will allow analysts to differentiate between bound and free clozapine (CLZ). This would enhance sensing characteristics such as specificity and performance through knowledge as to which components interact with the electrode surface, thereby offering an advantage over current TDM techniques.

In this work, we address protein-binding challenges for the specific case of electrochemically monitoring the antipsychotic CLZ. CLZ, a dibenzodiazepine that binds to serotonin and dopamine receptors prevalent throughout central and peripheral nervous tissue, is highly effective for managing treatment-resistant forms of schizophrenia [10,11]. CLZ is a drug where TDM is strongly recommended due to a well-established therapeutic range and potentially serious adverse effects [11,12]. These factors, and the burden to patients arising out of regular TDM, contribute to the underutilization of CLZ [12,13]. Due to its inherent electrochemical activity, i.e., its ability to participate in an electrochemical reaction, CLZ has become the target of miniaturized sensors aimed toward real-time, point of care (POC) monitoring of therapeutic concentrations in blood that could potentially alleviate the burden for patients and physicians [14].

Work from our group has demonstrated electrochemical sensing of CLZ with chitosan-based composite films in serum samples [3,6,15,16,17]. Unlike most previous studies, our approaches do not require sample pretreatment, which is typically employed to reduce interference and, similar to LC/MS procedures, often denatures and removes proteins [18]. The question remains though as to whether CLZ is detected in bound or unbound phases. While similar electrochemical (EC) sensors have been presented for other target analytes where this question also applies, no studies to date have addressed it. Here, we attempt to answer the question using a general approach, i.e., without the added variables from our biomaterial modifications, by employing unmodified glassy carbon electrodes and simulated solutions to isolate potential drug or drug-protein complex interactions with the electrode surface. 

Two proteins most likely to bind to drugs in blood are serum albumin (SA) and alpha-1 acid-glycoprotein (AAG), which possess several drug-specific binding sites that allow for hydrophobic and electrostatic interactions. While SA circulates at a higher concentration in the blood (~660 µM), AAG (~16 µM) is reported to have a higher affinity to CLZ [5,19,20]. Specifically, approximately 9.1% of CLZ binds to SA on site 2, also known as the benzodiazepine site. A further 84% of CLZ binds to the hydroxyl group on Tyr411 or Trp122 sites on AAG [21,22,23,24,25]. In pharmacology, the free drug concentration, as opposed to the total drug concentration, is suggested to reflect therapeutic efficacy in systemic circulation; exceptions occur, for example, when adequate equilibrium is not achievable without the bound fraction available, or in the case of haloperidol, when there is no correlation between the dosage and level of free fraction in circulation [26,27]. In the literature, CLZ does not present an exception, where the unbound form is arguably referred to as being “active” [21,22,28,29,30]. 

Using filtration to isolate the bound and free fractions, we investigated electrochemical oxidation signals obtained solely from unbound drug, specifically CLZ, after being mixed with individual solutions of bovine serum albumin (BSA) and human AAG in buffer. Our observations indicate that the electrochemical signals obtained measure the unbound form of drug in serum samples, with the presence of proteins attenuating the signal amplitude in the form of binding, fouling, and screening. This knowledge is critical in implementing TDM based on electrochemical sensors for appropriate dosage modifications in managing patient symptoms [27].

## 2. Results

### 2.1. Pure Species Redox Activity

**Figure 1:**
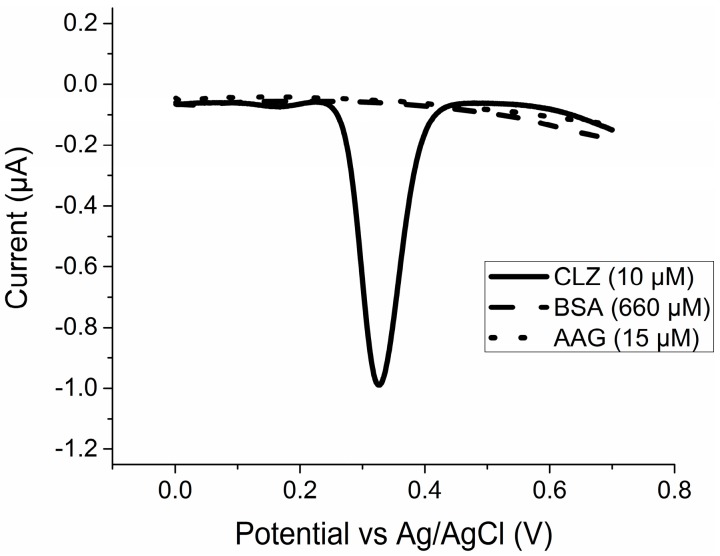
Differential pulse voltammograms for solutions containing BSA, AAG, or CLZ at 660, 15, and 10 µM, respectively.

Figure 1 displays the resulting voltammograms obtained using differential pulse voltammetry (DPV) from a CHI660D potentiostat (CH Instruments, Bee Cave, TX, USA) of individual species of interest, specifically CLZ, BSA, and AAG suspended in phosphate buffered saline (PBS). As demonstrated, the oxidation potential for peak current response from CLZ occurs at approximately 0.32 V. For 10 µM CLZ, this current response was approximately 0.99 µA. Current values near this peak potential range were observed during future measurements. No oxidation current peaks were observed from samples with only proteins, indicating the proteins do not react with the surface of the glassy carbon electrode (GCE) in their pure, unbound state. However, these measurements were taken of isolated species, necessitating the analysis of mixtures to measure a potential reaction between the bound protein–CLZ complex and the electrode.

### 2.2. Validation of Filter Functionality

Our mixtures of CLZ with either AAG or BSA contain unbound CLZ, unbound protein, or a fraction of CLZ–protein bound complexes. As shown in Figure 1, pure protein is not electrochemically active within our desired potential range compared to CLZ. To correlate the electrochemical signature of the mixed samples with free and/or complexed CLZ, free CLZ needed to be isolated from the solution. This was achieved through ultracentrifugation of the mixed solution through a 10 kDa-cutoff filter membrane. This allows passage of only small species such as unbound CLZ into the bottom reservoir, referred to as the filtrate. 

**Figure 2:**
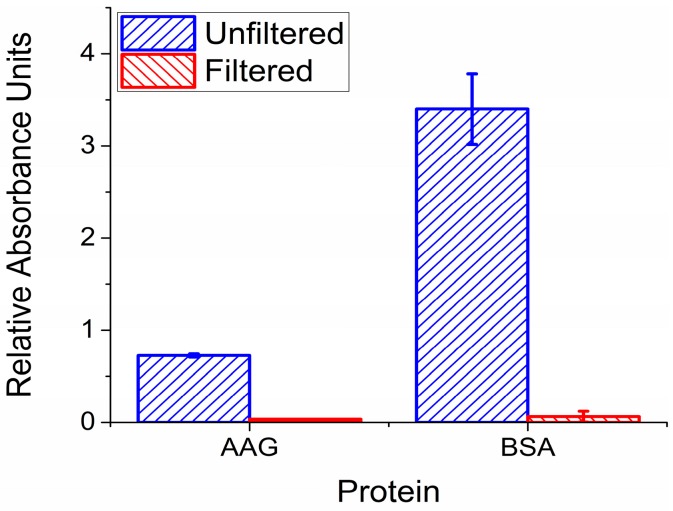
Relative absorbance of filtrate versus unfiltered solution containing protein in buffer. Low absorbance for the 100 μL AAG and BSA solution filtrates indicate effective protein retention. Filtrate values displayed as mean ± standard deviation, *n* = 3.

The first test to perform was to confirm the filter membranes would indeed prevent any protein from entering the filtrate during centrifugation. 100 μL solutions of 15 μM AAG or 660 μM BSA were ultracentrifuged through the membranes, and each absorbance was measured using ultraviolet (UV) spectroscopy for comparison. Relative absorbance of each solution for both AAG and BSA is displayed in Figure 2. Each filtrate is compared to measurements from an unfiltered solution that was distributed into three samples of equivalent volume. Compared to the PBS negative control, no significant amount of AAG passed the membrane into the filtrate. Our findings were further validated using MS, where proteins remained absent in the filtrates. This allowed us to determine with confidence that little to no protein binding occurred during electrochemical measurements of each filtrate. 

**Figure 3:**
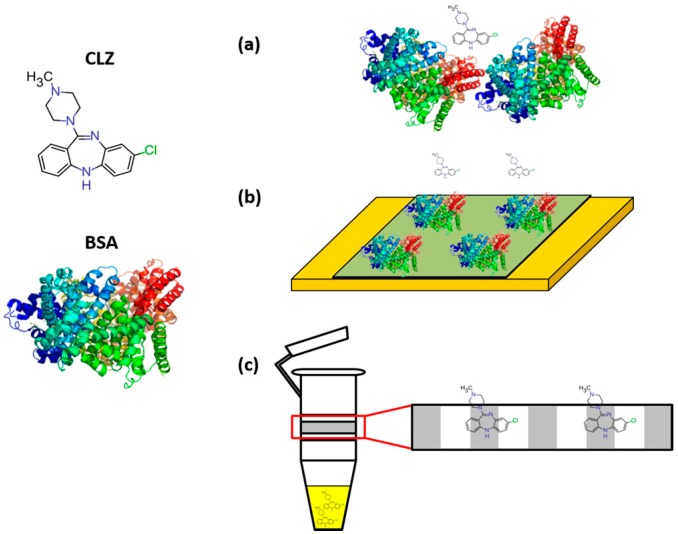
Schematics illustrating potential signal loss due to (**a**) screening, (**b**) electrode fouling and (**c**) non-specific membrane binding (NSMB). In (**a**), electrostatic interactions due to BSA crowding are preventing the CLZ from reaching the electrode entirely, whereas in (**b**), BSA aggregates directly onto the electrode surface, diminishing the exposed surface area for the CLZ to react with the electrode. On the left of (**c**), the CLZ is represented in the filtrate sample to be measured electrochemically, whereas the right side of the image depicts CLZ that has become trapped in the membrane during ultrafiltration.

The second test of filter functionality now considers CLZ, which would ideally pass through the membrane unhindered. In practice, it was necessary to determine how much CLZ is captured in the filter membrane to account for any signal loss, in this case referred to as non-specific membrane binding (NSMB) (Figure 3c), when performing DPV of the filtrate [24]. NSMB, calculated from Equation (1), is represented as an average percentage ± standard deviation across four samples for a range of CLZ concentrations in Table 1. Filtration membranes demonstrated significant levels of NSMB to CLZ, indicating it is necessary to be considered when normalizing filtrate concentrations. We did not observe a significant dependence of NSMB on CLZ (*p* > 0.05).

### 2.3. Quantification of Protein Fouling/Screening

While the proteins did not show inherent electrochemical activity, their presence could still affect the measurements indirectly through fouling or screening as shown in Figure 3b,c. Fouling refers to protein aggregation on the electrode surface, while screening refers to naturally occurring electrostatic interactions preventing analyte migration to the electrode. These must be considered as it becomes difficult to differentiate between a decreased CLZ signal due to protein–drug binding or due to screening/fouling. To investigate these effects, we used the model redox mediator FoFi because of its lack of affinity to BSA, so as not to form the bound complex that would have been formed with CLZ [31,32].

Fouling (Figure 3b) is represented as the percent signal reduction for the lowest concentration of FoFi used (5 μM), as this is the concentration that would be most impacted by fouling effects. The resulting fouling coefficients were 0.5 ± 0.6% and 1.8 ± 3.0% for AAG and BSA, respectively. As both signal reductions were negligible, fouling was neglected in our subsequent analysis.

The impact of screening (Figure 3a) proved more significant for BSA as presented in Table 2 across varying concentrations of FoFi, reflecting the concentrations of CLZ employed later. The low concentration of AAG, on the other hand, did not impact the reaction between the analyte and the electrode as drastically, reducing the signal by only 8.6 ± 2.3%. The signal reduction from BSA was expected to be greater than that of the AAG, as it is both a larger molecule and in a higher concentration. Signal reductions less than 10% were judged to be insignificant and were neglected in subsequent analysis. 

### 2.4. Unfiltered vs. Filtrate Comparison

**Figure 4:**
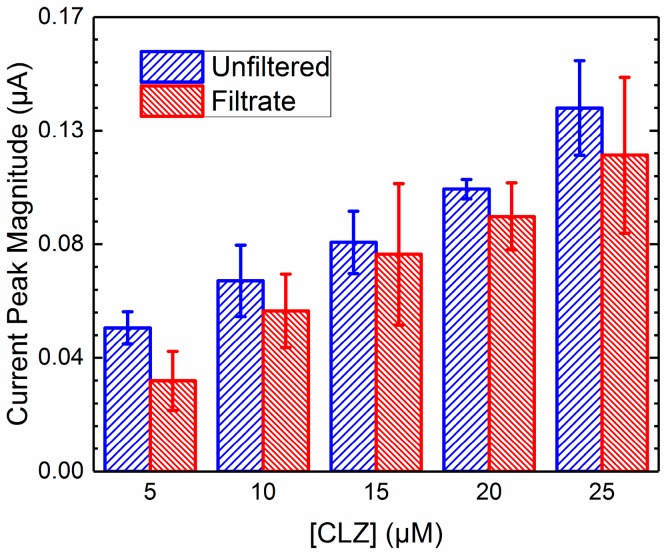
Current peak magnitudes of unfiltered solutions and filtrates of AAG and CLZ mixtures across varying concentrations of CLZ. Values displayed as mean ± standard deviation; *p* = 0.90 between unfiltered vs. filtrate groups, *n* = 3.

**Figure 5:**
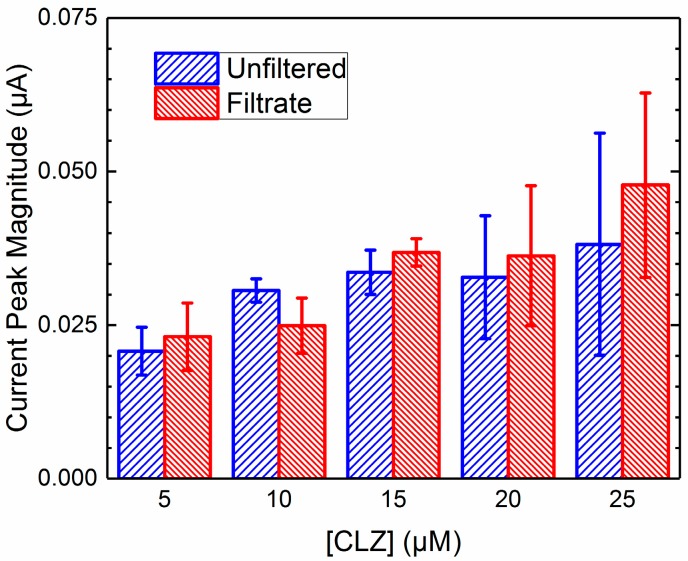
Current peak magnitudes of unfiltered solutions and filtrates of BSA and CLZ mixtures across varying concentrations of CLZ. Values displayed as mean ± standard deviation; *p* = 0.73 between unfiltered vs. filtrate groups, *n* = 3.

In order to compare the effective activity of the CLZ in the filtrate to the electroactive species in the mixture, it is necessary to account for any signal loss due to screening, fouling, and nonspecific membrane binding, illustrated in Figure 3. The electrochemical data from mixtures was normalized using values from Table 1 to account for NSMB of the CLZ to the filter membrane for signal loss of the filtrate, and values from Table 2 to account for the screening due to BSA in the unfiltered mixed solution. Due to the negligible impact of fouling from both proteins and screening from AAG, these factors were disregarded. Sets of data were generated across each concentration of CLZ for unfiltered mixtures and the resulting filtrates from the ultrafiltration procedures; these sets are displayed in Figure 4 and Figure 5 for AAG and BSA, respectively. Resulting *p*-values from two-way ANOVA (α = 0.05) were 0.90 and 0.73, indicating no significant difference between the data sets.

Using data from pure CLZ solutions and Equation (2) in Section 4.4, we calculated the percent protein binding for both AAG and BSA for each concentration of CLZ. The results are presented in Table 3 below. High intra-assay variability resulted likely due to differences in the filter membranes utilized or to the significant sensitivity to light exhibited by CLZ. Resulting *p*-values from 2-way ANOVA (α = 0.05) were 0.92 and 0.14 for AAG and BSA, respectively, indicating no significant difference in binding with increasing concentrations of CLZ.

## 3. Discussion

Overall, our experiments present the first pieces of evidence that the electrochemical oxidation of free CLZ constitutes the vast majority, if not all, of the activity measured in the unfiltered mixed solution. Thus, the activity of CLZ–protein complexes is more characteristic of pure protein than of pure drug, indicating blood proteins block active functional groups from drugs of this size and hinder reactions with the electrode [19,21,22].

Our results in Section 2.4 allow us to deduce that the percent signal reduction of the mixed solutions compared to pure samples of CLZ at the same concentration is proportional to the ratio of CLZ that has become bound to the proteins. Table 3 presents this ratio as percent protein binding for both AAG and BSA. Values did not vary significantly across increasing CLZ concentrations, though AAG was at a significantly lower concentration indicating there is an elevated affinity between CLZ and AAG (16 µM) than between CLZ and BSA (660 µM). Mean percent binding for CLZ in isolation with AAG and BSA were 30.5% and 72.7%, respectively, indicating characteristic affinities of CLZ to these proteins in isolation. These values may vary in serum due to the occurrence of competitive binding, as both proteins are present in addition to other species. While the percent binding of AAG and BSA were comparable at approximately a 1:2 ratio, the concentration of BSA was 44 times greater than AAG, used to reflect concentrations similar to physiological values and demonstrating the stronger binding potential between CLZ and AAG [24,25]. 

The implications of our results for an EC-CLZ sensor are twofold. First, with EC sensitive mainly to the free CLZ, it offers a much more direct route to access this information compared to LC/MS, where additional pretreatment is required. This indicates that the resulting signal, the unbound CLZ, may provide critical information in understanding the efficacy of a drug in circulation [21,27]; Second, EC sensing in combination with sample pretreatment to unbind drug/protein complexes will significantly enhance the signal-to-noise ratio—in part through eliminating fouling and screening, but mostly due to liberating the total available CLZ for EC measurement. This can be achieved by various techniques, such as applying increased temperatures, a change in pH, alcohol, or proteases [33,34,35,36].

We note that our analysis assumes that enzymolysis of the CLZ from the proteins used does not occur, as it does not contain the functional groups required [37,38]. Several forms of potential electrochemical interference from drugs that we do not address in this work are those from metabolic byproducts and matrix effects from blood or serum. Common metabolites of CLZ are *N*-desmethylclozapine and clozapine N-oxide; the unbound fractions of each of these species have been shown to be consistent in serum, regardless of concentration [39]. In our previous work, we have used a catechol-modified chitosan electrode system to differentiate electrochemical signals between clozapine and *N*-desmethylclozapine, as they are both pharmacologically active but the relative reduction potentials are differentiated in the demonstrated system [3,14]. Though N-oxide has been shown to bind heavily to proteins, its clinical relevance remains controversial and thus was not pursued in this study [12,39]. On the other hand, the heterogeneity of biological samples such as blood or serum often induces a variety of matrix effects, such as altering analyte ionization, that significantly alter analytical results and must be controlled [40]. There is potential to expand upon this study to determine additional causes of this change in electrochemical signal, though the results presented in this study indicate a strong influence of protein-binding and its variability across various serum proteins that must be addressed for effective species identification of electrochemical events.

## 4. Materials and Methods 

### 4.1. Chemicals

All reagents were obtained in crystalline form from Sigma (St. Louis, MO, USA). Human AAG (44 kDa) and BSA (66 kDa) were dissolved in 1× phosphate buffered saline (PBS: NaCl 13.8 M; KCl 0.27 M, Sigma) at constant concentrations of 15 μM and 660 μM, respectively, to reflect average concentrations in blood [2]. Clozapine (330 Da) was dissolved in methanol at 10 mm, then spiked into the PBS-based sample solutions at 5, 10, 15, 20, and 25 μM. Mixing at approximately 200 rpm for 1 h at room temperature induced binding between CLZ and each protein.

### 4.2. Free CLZ Isolation—Filtration

Cellulose filter membranes (Merck Millipore, Billerica, MA, USA) were used to remove protein-bound complexes and free protein from solutions with a 10 kDa cutoff. Filtration was performed using a Jouan CR3i centrifuge (Thermo Fisher, Waltham, MA, USA). Filters were rinsed at 14,000× *g* for 20 min with deionized (DI) water (>16 MΩ cm) to remove glycerine residues in the membrane. Filters were then filled with 500 μL of solution containing either CLZ only or CLZ- and AAG, while 100 μL was used for CLZ-BSA mixtures due to filter requirements. Samples were centrifuged at 14,000× *g* for 40 min, and solutions were subsequently removed from the lower compartment, referred to as the filtrate, for analysis. 

### 4.3. Ultraviolet Visible Spectroscopy

Each sample (50 μL) was transferred to a UV-transparent 96-well Microtiter plate (Sigma), both before filtration and from the resulting filtrate, and absorbance spectra recorded using a SpectraMax Plus 384 (Molecular Devices, Sunnyvale, CA, USA). Readings were taken at wavelengths of 200–350 nm in 5 nm increments. Analysis to control for nonspecific membrane binding (NSMB) of free CLZ to the filter membrane was performed by comparing the filtrate absorbance to that of the unfiltered solution at 205 nm wavelength. Calculations for NSMB were performed using Equation (1),

(1) $$NSMB=\left(\frac{C_{drug\;total}-C_{drug\;filtrate}}{C_{drug\;total}}\right)$$

where *C_drug total_* is the donor (total) drug concentration in PBS before centrifugation, and *C_drug filtrate_* is the drug concentration in the PBS filtrate after centrifugation. The molar absorptivity, 0.0027 L mol^−1^ cm^−1^, was calculated using Beer’s law by taking the slope of the line of best fit from the concentration vs. absorbance plot.

Values for concentration were obtained from the absorbance measurements, as they are proportional to each other, and were implemented in Equation (1). Analysis was performed using Microsoft Excel and MathWorks MATLAB (R2013b).

### 4.4. Percentage Protein Binding

The percentage of CLZ bound (PB) to either protein at each concentration was calculated using Equation (2),

(2) $$PB=100\;\times \;\left(1-\frac{C_{mix\;filtrate}}{\left(1-NSMB\right)\;\times \;C_{drug\;total}}\right)$$

where *C_mix filtrate_* is the concentration of CLZ in the filtrate of the protein-CLZ mixture, reflected in the spectroscopic absorbance.

### 4.5. Electrochemistry

All electrochemistry was performed using a CHI660D potentiostat with a standard three-electrode setup, consisting of a glassy-carbon working electrode (GCE, 3 mm diameter), a platinum wire counter electrode (2 mm diameter), and a Ag/AgCl reference electrode (1 M KCl), all from CH Instruments (Bee Cave, TX, USA). Prior to each experiment, each GCE was polished and standardized validation was performed using a ferrocyanide/ferricyanide (FoFi: 1 M phosphate buffer saline, 5 mm ferricyanide, 5 mm ferrocyanide) redox couple using cyclic voltammetry (CV: initial *E* and final *E* = 0.19 V, high *E* = 0.44 V, low *E* = −0.06 V, scan rate = 0.2 V/s, 3 sweep cycles). Differential pulse voltammetry (DPV: initial *E* = 0 V, final *E* = 0.7 V, incremental *E* = 0.001 V, amplitude = 0.05 V, pulse width = 0.2 s, sampling width = 0.0167 V/s, pulse period = 0.5 s) was implemented for each unfiltered sample and corresponding filtrate. The above experiments were all performed in triplicate and analysis was performed with Microsoft Excel and OriginLab OriginPro.

### 4.6. Mass Spectrometry

Mass spectrometry (MS) was used to verify that the filter membrane actually prevents proteins from entering the filtrate during centrifugation. MS was performed comparing prefiltered samples of 660 μM BSA and its corresponding filtrate from the centrifugation method above using an AccuTOF-CS (JEOL, Peabody, MA, USA), which implements electrospray ionization as a standard. Samples were dissolved in 1:1 methanol: DIH_2_O. Positive mode was used with a mass measurement accuracy of <5 ppm. Tool parameters used were as follows: needle voltage = 2100 V, orifice #1 voltage = 30 V, ring voltage = 10 V, orifice #2 voltage = 5 V, desolvating chamber temperature = 250 °C, orifice #1 temperature = 100 °C, and mass range = 300–3000 m/z.

### 4.7. Electrode Fouling/Screening

The activity of a 5 μM ferrocene dimethanol (Fc) solution in PBS was measured using the DPV parameters described above, followed by a solution of 15 μM AAG (or 660 μM BSA), then a 5 μM Fc solution measurement again without polishing the electrode between runs to observe any differences in the Fc signal amplitude. Any decrease in signal would correspond to signal impedance due to fouling of the electrode surface. Fc was used as it is redox capable but would eliminate the potential for the signal to diminish due to binding [41].

Screening tests entailed using FoFi solutions at concentrations comparable to CLZ while mixed with physiologic concentrations of each protein. CV (initial E and final E = 0.19 V, high E = 0.44 V, low *E* = −0.06 V, scan rate = 0.01 V/s, 15 sweep segments,) was performed first on solutions of FoFi at 5 to 25 μM concentrations at 5 μM increments, then on solutions with the same concentrations but with added BSA at 660 μM. All screening experiments were performed in duplicate and analyses were performed in Microsoft Excel.

## 5. Conclusions

In this study, we investigate phenomena that need to be addressed in achieving a thorough TDM strategy. Specifically, we effectively isolated an electrochemically active analyte from a solution that included free proteins and indirectly quantify the concentration of protein-bound analyte after approximating the percent of bound CLZ. Not only does this help remove larger interfering species relative to the membrane cutoff, this can be replicated with other smaller analytes that interact similarly with larger molecules. In our study, the challenge of determining NSMB and preventing the leakage of proteins into the filtrate are addressed. When discussing isolation of other species from their bound complexes, equilibrium dialysis is often used as a gold standard; however, it must be performed at a specific temperature, and the stability of the drug or protein at that temperature must be understood [42].

We present several observations in this study. Our major finding from these experiments is the lack of significant electrochemical activity resulting from CLZ–protein bound complexes. Since nearly all activity is due to the unbound drug, we can infer that the complex is not active. Therefore, it is possible to treat the protein-bound complex as merely an interfering species that diminishes the electrochemical oxidation signal in the measured solution. The use of unmodified electrodes and pure species in buffer in this work was required to isolate specific activity between the protein-bound complexes and the electrode. These findings offer potential to electrochemically distinguish between bound and unbound phases of CLZ to contribute to our previous efforts detecting CLZ in serum samples using chitosan-based electrode modification [3,6,14,15,16,17].

In conclusion, we present data valuable to TDM strategies by addressing the activity of electrochemical species when they are bound to serum proteins, overall offering measurements that will contribute toward the selectivity of a CLZ sensor currently under development [15]. 

## Figures and Tables

**Table 1:** NSMB calculated for varying CLZ concentrations. Values displayed as mean ± standard deviation (*n* = 4).

[CLZ] (μM)	NSMB (%)
5	18.7 ± 5.5
10	26.9 ± 7.1
15	27.9 ± 6.3
20	17.3 ± 11.2
25	30.2 ± 16.9

**Table 2:** Coefficients for signal loss (%) from protein (BSA) screening across varying FoFi concentrations. Values displayed as mean ± standard deviation (*n* = 2).

[FoFi] (μM)	Signal Reduction Due to Screening (%)
5	1.7 ± 6.0
10	25.7 ± 4.7
15	14.7 ± 3.0
20	16.0 ± 21.0
25	7.1 ± 2.2

**Table 3:** Percentage protein binding for both AAG and BSA (normalized for screening from the fouling coefficients) across varying concentrations of CLZ. Values displayed as mean ± standard deviation *n* = 3.

[CLZ] (μM)	Binding to AAG (%)	Binding to BSA (%)
5	36.8 ± 7.1	73.2 ± 5.0
10	29.8 ± 13.1	67.0 ± 2.0
15	28.7 ± 9.7	69.4 ± 3.3
20	31.4 ± 2.4	76.7 ± 7.1
25	25.6 ± 9.7	77.1 ± 10.8
